# Prognostic significance of COVID-19 MSCT chest findings on short-term disease progression

**DOI:** 10.1186/s43168-022-00136-8

**Published:** 2022-05-31

**Authors:** Mohamed H. Faheem, Amr Gomaa, Amira H. Allam

**Affiliations:** 1 Department of Radio-diagnosis, Faculty of Medicine, Benha University, Benha, Egypt; 2 Department of Chest Diseases, Faculty of Medicine, Benha University, Benha, 13512 Egypt

**Keywords:** COVID-19, MSCT, Chest, Corona

## Abstract

**Background:**

CT has been used on a massive scale to help identify and investigate suspected or confirmed cases of COVID-19 pneumonia. This study aimed to assess the prognostic significance of the chest findings MSCT of COVID-19 patients and to determine if prognosis can rely on the initial CT imaging.

**Methods:**

The study design was retrospective cohort study. It was carried out on 300 patients presented to the chest outpatient clinics in Benha University hospitals and El Abbassia Chest Hospital with clinical picture suggestive of COVID-19 infection. The CT finding were then compared to the short-term clinical outcome of the patients (1–3 weeks), acquired from the hospital patient data archive. According to the progression of the respiratory symptoms (including dyspnea, respiratory rate, and O2 saturation), the short-term clinical outcome of the patients was classified into 4 groups: group A (mild cases), group B (moderate cases), group C (severe cases), and group D (fatality cases).

**Results:**

Consolidations, septal thickening, crazy paving, and fibrotic bands were significantly higher in groups C and D than group A and B (*P*-value < 0.001 for all variants). Nodules show statistically significant higher incidence in groups A and B than group C and D (*P*-value < 0.001). The CT severity score shows statistically significant increase with the poor short-term clinical outcomes (groups C and D) (*P*-value < 0.001).

**Conclusion:**

CT chest is a good radiological marker that can help in predicting short-term clinical outcome in COVID-19 patient. Higher CT severity scores are predictors of poorer clinical prognosis.

## Introduction

The 2019 novel coronavirus (2019-nCoV), initially reported in Wuhan, China, has been declared a global health emergency by the World Health Organization [1].

Although reverse transcriptase polymerase chain reaction (RT-PCR) remains the standard diagnostic reference for COVID-19 infection, the high false-negative rate and its restricted availability limit the prompt diagnosis, which can result in a growing number of cases given the contagiousness of the virus [2]. So, CT has been used on a massive scale to help identify and investigate suspected or confirmed cases of COVID-19 [3].

Imaging provides the healthcare facilities with a feasible screening tool for the detection of early pneumonia in high-risk patients. It also aids the physicians in the diagnosis before the RT-PCR results get available. Last but not least, it could be useful in assessing the severity and course of disease progression [2].

According to current experience, lung CT imaging may manifest abnormalities earlier than RT-PCR testing. Recently performed studies suggest that CT could have higher sensitivity than RT-PCR in the diagnosis of COVID-19-associated pneumonia. Currently, high-resolution CT has been included as one of the main tools for screening, primary diagnosis, and evaluation of disease severity [4].

Multiple recent studies from China and South Korea revealed that ground-glass opacities (GGO) are the most common finding in COVID-19-associated pneumonia. Both lungs are involved in almost all cases, and a peripheral subpleural distribution is the most reported location. Multiple other radiologic features have also been described, although imaging features are highly nonspecific [5].

Few studies investigated the short-term prognostic values of the chest CT findings, e.g., that critically ill patients had more consolidation pattern, central involvement of the lungs (peri-bronchovascular), developed air bronchograms, and those who had more crazy paving appearance and pleural effusion, findings which may reflect the virulence of COVID-19. Therefore, these radiologic features have the potential to represent prognostic imaging markers in patients with COVID-19 pneumonia [6].

This study aimed to assess the prognostic significance of the chest findings of MSCT of COVID-19 patients and to determine if prognosis can rely on the initial CT imaging.

## Patients and methods

The study design was a retrospective cohort study. It was carried out on 300 patients presented to the chest outpatient clinics in Benha University hospitals and El Abbassia Chest Hospital with clinical picture suggestive of COVID-19 infection during the period from April 2020 to December 2020.

### Inclusion criteria

- Patients who had positive RT-PCR test for COVID-19 infection and show positive chest CT findings.

### Exclusion criteria

- Patients with negative RT-PCR test for COVID-19 infection
- Patients with positive RT-PCR test for COVID-19 and negative chest CT findings
- Patients with other pre-existing lung pathologies in the chest CT

Data- which were collected from patient' files - included the following: history including symptoms and comorbidities and previously available examination data.

Approval was taken from the ethics committee of researchers of Benha University. An informed consent was taken from all the participants.

The consent contains the following:

1. Their approval that their studies and clinical data could be used for research purpose
2. All patients’ clinical data is considered confidential.
3. Signatures or fingerprints of the participants were taken.

The picture archiving and communication systems (PACS) in both hospitals were revised during the period from the 1st of April 2020 to the 31st of December 2020. The included CT studies were revised first for technical appropriateness, and the studies with technical problems were excluded.

All chest CT examinations were performed using multislice CT equipment (Toshiba Activion 16 and Toshiba Alexion 16). The exams were performed with patients in supine position and breath-hold during full expiration. The scanning range was from the lower neck down to the level of the adrenal glands. The scanning parameters were as follows: helical scanning mode; tube voltage, 120 kV; tube current–time product, 50–350 mAs; pitch, 1.2 and 1.375; matrix, 512 × 512; slice thickness, 5 mm; reconstructed in lung window; and reconstructed slice thickness, 1.25 mm, followed by sagittal and coronal reconstruction.

The technically accepted studies were extracted from the PACS system. They were interpreted by radiologists and pulmonologist (14 and 10 years in chest CT interpretation respectively) in blind manner.

The following CT features were assessed: distribution (peripheral, central, or central and peripheral), number of lobes involved (one, two or three, four or five), shape (patchy, nodular), appearance (ground-glass opacity [GGO], consolidation, or GGO with consolidation), specific signs within the lesions (vascular thickening, crazy paving pattern, air bronchogram sign, halo sign, and fibrosis), size of largest lesion (< 1 cm, 1–3 cm, > 3 cm), and extrapulmonary manifestations (mediastinal and hilar lymph node enlargement, pleural effusion, pleural thickening). CT severity scoring was calculated for each case [2].

The CT finding was then compared to the short-term clinical outcome of the patients (1–3 weeks), acquired from the hospital patient data archive. According to the progression of the respiratory symptoms (dyspnea, respiratory rate, and O2 saturation), the short-term clinical outcome of patients was classified into 4 groups, as follows:

1. Group A (mild cases): with no progression of the respiratory symptoms
2. Group B (moderate cases): who have worsened disease but not requiring ICU admission
3. Group C (severe cases): patient who needed ICU admission
4. Group D (fatality cases): cases who died with or without ICU admission.

## Statistical analysis

Data were collected, revised, coded, and entered to the Statistical Package for Social Science (IBM SPSS) version 20. The qualitative data were presented as number and percentages, while quantitative data were presented as mean, standard deviations, and ranges when their distribution is found parametric. The comparison between two groups with qualitative data was done by using chi-square test and/or Fisher exact test which was used instead of chi-square test when the expected count in any cell was found less than 5. The comparison between more than two independent groups with quantitative data and parametric distribution was done by using one-way ANOVA test. The confidence interval was set to 95%, and the margin of error accepted was set to 5%. So, the *P*-value was considered significant as the following: *p* > 0.05 = non-significant (NS), *P* < 0.05 = significant (S), and *P* < 0.001 = highly significant (HS)

## Results

This study was a retrospective cohort study including the patients presented to the chest outpatient clinics in Benha University hospitals and El Abbassia Chest Hospital with clinical picture suggestive of COVID-19 infection, during the period from April 2020 to December 2020. The total number of the study sample is 300 patients. One-hundred fifty-two (50.7%) were females, while 148 (49.3%) were males. The mean age of the study population was 54.24 years with standard deviation of 13.5 years. Regarding clinical outcomes, 75 (25%) were within group A (mild cases), 125 (41.7%) group B (moderate cases), 85 (28.3 %) group C (marked cases), while 15 (5%) were within group D (fatality cases) (Table 1).

**Table 1:** Distribution of the studied cases according to clinical outcomes, sex, and age

		No. = 300
**Clinical outcomes**	Group A	75 (25.0%)
	Group B	125 (41.7%)
	Group C	85 (28.3%)
	Group D	15 (5.0%)
**Sex**	Female	152 (50.7%)
	Male	148 (49.3%)
**Age**	Mean ± SD	54.24 ± 13.51
	Range	20–90

Consolidations, septal thickening, crazy paving, and fibrotic bands showed significant statistical difference between groups C and D and groups A and B (*P*-value < 0.001 for all variants). Nodules were statistically higher in groups A and B than groups C and D (*P*-value < 0.001) (Table 2).

**Table 2:** Comparison between group A (no. = 75), group B (no. = 125), group C (no. = 85), and group D (no. = 15) regarding findings

Findings	Group A		Group B		Group C		Group D		Test value*	*p*-value
	No.	%	No.	%	No.	%	No.	%		
**GGO**	75	100.0%	125	100.0%	85	100.0%	15	100.0%	NA	NA
**Consolidations**	5	6.7%	30	24.0%	38	44.7%	13	86.7%	54.452	0.001
**Nodules**	23	30.7%	18	14.4%	5	5.9%	2	13.3%	18.796	0.001
**Septal thickening**	35	46.7%	95	76.0%	82	96.5%	15	100.0%	59.069	0.001
**Crazy pavings**	0	0.0%	3	2.4%	19	22.4%	10	66.7%	79.468	0.001
**Fibrotic bands**	14	18.7%	55	44.0%	67	78.8%	15	100.0%	74.490	0.001
**Lymphadenopathy**	0	0.0%	0	0.0%	3	3.5%	0	0.0%	7.665	0.043

*P*-value > 0.05, nonsignificant (NS); *P*-value < 0.05, significant (S); *P*-value < 0.01, highly significant (HS). *Chi-square test, •one-way ANOVA test

The unilateral distribution occurs significantly higher in group A compared to the other groups (*P*-value < 0.001) (Table 3).

**Table 3:** Comparison between group A (no. = 75), group B (no. = 125), group C (no. = 85), and group D (no. = 15) regarding site

Site	Group A		Group B		Group C		Group D		Test value*	*p*-value
	No.	%	No.	%	No.	%	No.	%		
**Unilateral**	10	13.3%	1	0.8%	0	0.0%	0	0.0%	26.556	0.001
**Bilateral**	65	86.7%	124	99.2%	85	100.0%	15	100.0%		

*P*-value > 0.05, nonsignificant (NS); *P*-value < 0.05, significant (S); *P*-value < 0.01, highly significant (HS). *Chi-square test, •one-way ANOVA test

The lobar predominance was statistically different between different groups (*P*-value < 0.001). No lobar predominance occurs significantly higher in group C compared to the other groups (*P*-value < 0.001) (Table 4).

**Table 4:** Comparison between group A (no. = 75), group B (no. = 125), group C (no. = 85), and group D (no. = 15) regarding lobar predominance

Lobar predominance	Group A		Group B		Group C		Group D		Test value*	*p*-value
	No.	%	No.	%	No.	%	No.	%		
**Lower**	22	29.3%	18	14.4%	9	10.6%	5	33.3%	13.177	0.001
**Upper**	0	0.0%	0	0.0%	0	0.0%	0	0.0%		
**No**	53	70.7%	107	85.6%	76	89.4%	10	66.7%		

*P*-value > 0.05, nonsignificant (NS); *P*-value < 0.05, significant(S); *P*-value < 0.01, highly significant (HS). *Chi-square test, •one-way ANOVA test

The confluent lesions occurred significantly higher in groups C and D compared to groups A and B (*P*-value < 0.001) (Table 5).The CT severity score was statistically higher in patients with the poor short-term clinical outcomes (groups C and D) (*P*-value < 0.001) (Table 6).

**Table 5:** Comparison between group A (no. = 75), group B (no. = 125), group C (no. = 85), and group D (no. = 15) regarding centrality

Centrality	Group A		Group B		Group C		Group D		Test value*	*p*-value
	No.	%	No.	%	No.	%	No.	%		
**Peripheral**	75	100.0%	121	96.8%	52	61.2%	1	6.7%	122.866	0.001
**Central**	0	0.0%	0	0.0%	0	0.0%	0	0.0%		
**Confluent**	0	0.0%	4	3.2%	33	38.8%	14	93.3%		

*P*-value > 0.05, nonsignificant (NS); *P*-value < 0.05, significant (S); *P*-value < 0.01, highly significant (HS). *Chi-square test, •one-way ANOVA test

**Table 6:** Comparison between group A (no. = 75), group B (no. = 125), group C (no. = 85), and group D (no. = 15) regarding CT severity score

CT severity score	Group A	Group B	Group C	Group D	Test value	*p*-value
	No. = 75	No. = 125	No. = 85	No. = 15		
**Minimal**	39 (52.0%)	5 (4.0%)	0 (0.0%)	0 (0.0%)	311.028	0.001
**Mild**	36 (48.0%)	77 (61.6%)	7 (8.2%)	0 (0.0%)		
**Moderate**	0 (0.0%)	42 (33.6%)	61 (71.8%)	2 (13.3%)		
**Marked**	0 (0.0%)	1 (0.8%)	17 (20.0%)	13 (86.7%)		
**Mean ± SD**	6.69 ± 2.81	11.67 ± 2.93	16.48 ± 2.66	20.53 ± 1.96	210.740	0.001
**Range**	1–12	2–21	10–22	17–23		

*P*-value > 0.05, nonsignificant (NS); *P*-value < 0.05, significant(S); *P*-value < 0.01, highly significant (HS). *Chi-square test, •one-way ANOVA test

### Illustrative cases

#### Case 1

**Fig. 1:**
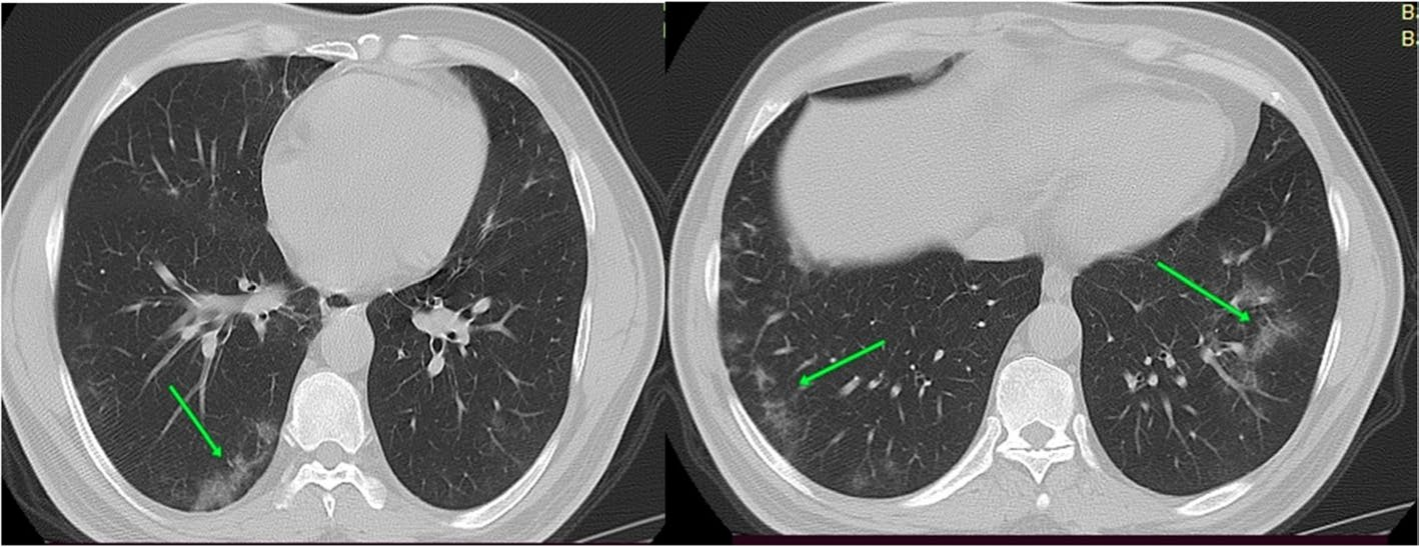
CT chest axial cuts with sagittal and coronal reformatted images were done with no preparations. CT findings were bilateral ground-glass densities with septal thickening and peripheral predominance. Axial cuts CT interpretations suggestive of atypical viral pneumonia (COVID-19) (CORAD 5) with CT severity score = 11 (mild affection). By following the patient after 3 weeks, the symptoms became worse but no need for hospitalization (group B)

Male patient aged 48 years presented by cough, dyspnea, and mild fever. Laboratory investigations revealed increase CRP and lymphopenia (Fig. 1).

#### Case 2

**Fig. 2:**
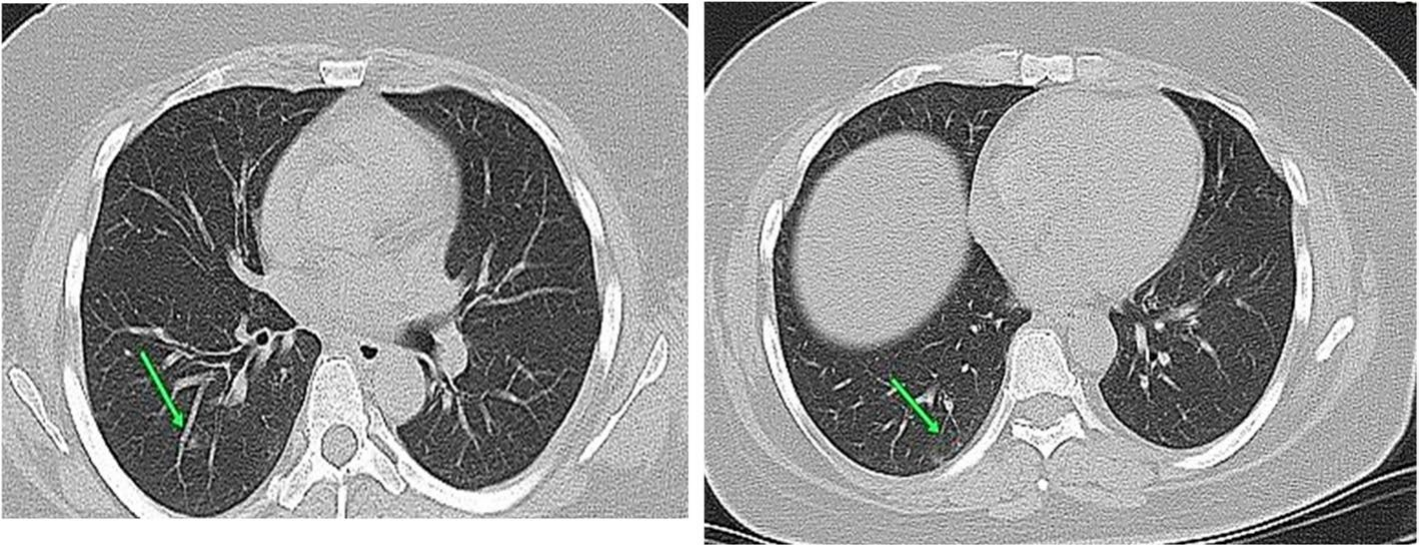
CT chest axial cuts with sagittal and coronal reformatted images were done with no preparations. CT findings were unilateral ground-glass densities with peripheral predominance (arrowed). Axial CT cuts; CT interpretations suggestive of atypical viral pneumonia (COVID-19) (CO-RAD 3) with CT severity score = 4 (minimal affection). By following the patient after 3 weeks, no progression of the symptoms and no need for hospitalization (group A)

Female patient aged 50 years presented by fever. Laboratory investigations revealed lymphopenia (Fig. 2).

#### Case 3

**Fig. 3:**
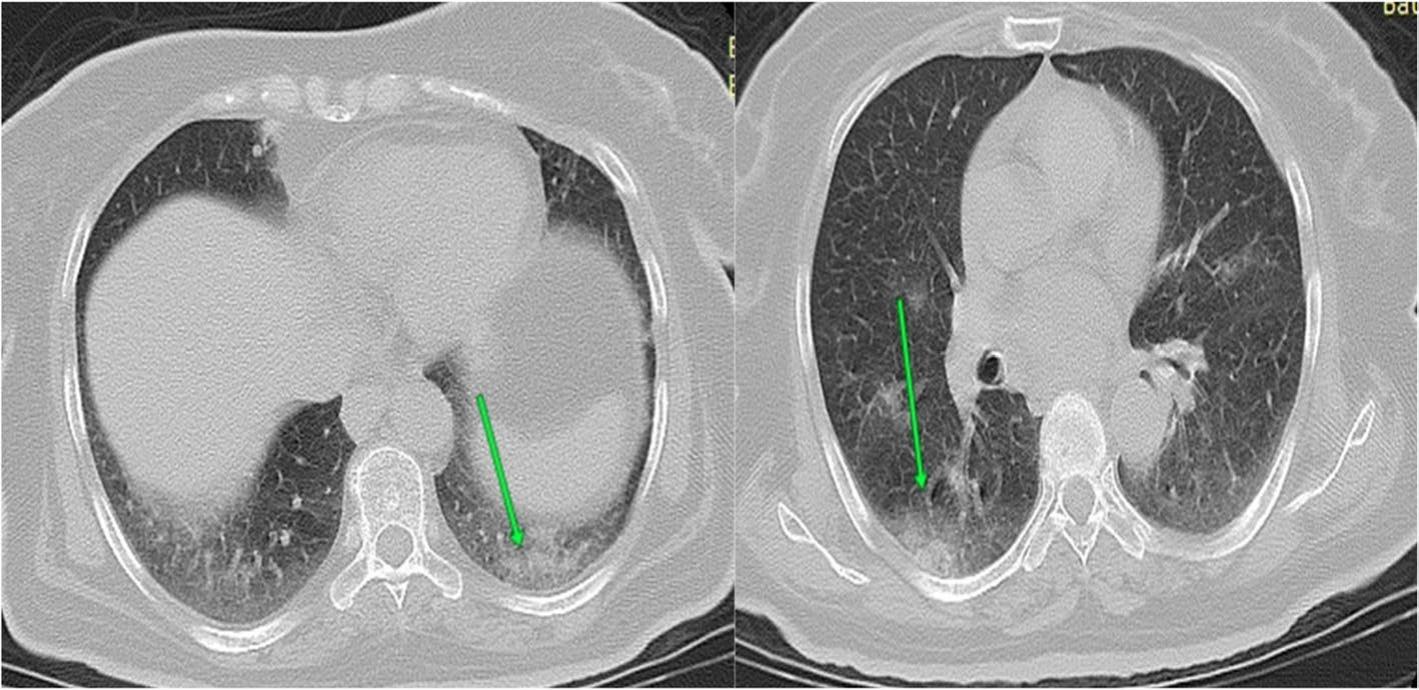
CT chest axial cuts with sagittal and coronal reformatted images were done with no preparations. CT findings were bilateral ground glass/consolidation densities with peripheral predominance and septal thickening as well as fibrotic bands (arrowed). Axial CT cuts; CT interpretations suggestive of atypical viral pneumonia (COVID-19) (CO-RAD 5) with CT severity score = 17 (moderate affection). By following the patient after 3 weeks, the symptoms became worse and need hospitalization followed by ICU admission (group C)

Female patient aged 72 years presented by severe cough, dyspnea, and severe fever. Laboratory investigations revealed lymphopenia and +ve PCR (Fig. 3).

#### Case 4

**Fig. 4:**
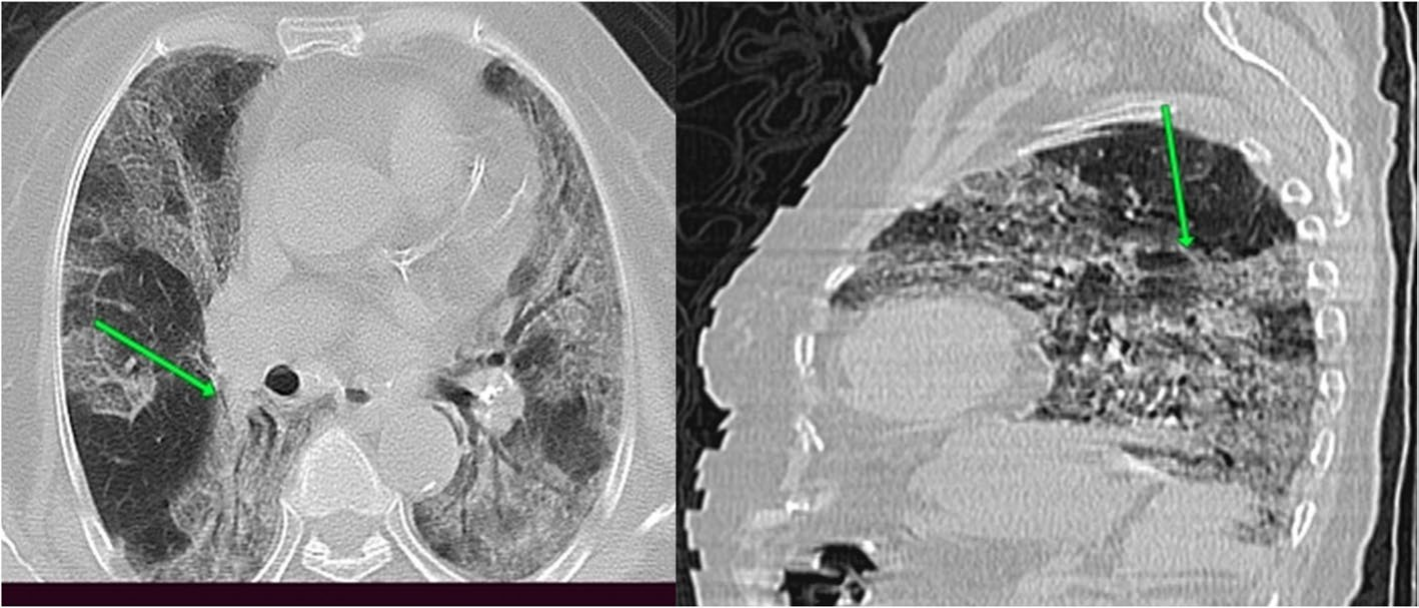
CT chest axial cuts with sagittal and coronal reformatted images were done with no preparations. CT findings were confluent bilateral ground-glass/consolidation densities with septal thickening, fibrotic bands, and crazy paving appearance (arrowed). Axial and sagittal CT cuts; CT interpretations suggestive of atypical viral pneumonia (COVID-19) (CO-RAD 5) with CT severity score = 23 (marked affection). By following the patient after 3 weeks, the patient died after ICU admission (group D)

Female patient aged 88 years presented by severe symptoms in the form of cough, dyspnea, fever, and cyanosis. Laboratory investigations revealed increase CRP and lymphopenia as well as +ve PCR (Fig. 4).

## Discussion

The purpose of this study was to assess the short-term prognostic value of the CT chest findings in COVID-19 patients and whether we can rely on the initial CT findings in predicting the prognosis.

Among 300 patients included in this study, 148 were males and 152 were females. The range of age in this study sample was 20–90 years with mean equals 52.51+/−13.56. Haji Ahmadi et al. found significant increase in the ICU admission and mortality with increasing age, decreasing O2 saturation in patients with COVID-19 pneumonia [7].

In the current study, as regard the short-term clinical outcomes, 75 cases were within group A (mild cases), 125 cases were within group B (moderate cases), 85 cases were within group C (marked cases), while 15 cases were within group D (fatality cases). The distribution of the clinical outcome within the sample of Tabatabaei et al. study was different. They reported that 96 of 120 patients were hospitalized in the routine ward. Eleven patients were admitted to the ICU and intubated, whereas 13 patients were died [6].

In the current study, 164 of 300 patients were seen with minimal to mild CT involvement, while 104 patients showing moderate involvement and only 31 patients show marked involvement. The presence of GGOs only was usually observed in cases with minimal and mild CT scoring; however, consolidative areas and crazy paving were seen in cases with moderate and marked CT scoring. Morbidity and mortality were lower in patients with minimal, mild, and moderate CT affection. However, patients with marked CT affection had higher morbidity and mortality. This matches with Hefeda (2020) study which stated that GGO is the most common and the earliest sign of COVID-19 pneumonia, high mortality in patients with consolidation. It is declared also that consolidation and crazy paving are more common in patients with severe or advanced disease [8]. Chest CT images could manifest different imaging features or patterns in COVID-19 patients with a different time course and disease severity [9].

This study stated that bilateral affection was the most common pattern. This matches with what was concluded by Zheng Ye et al. [9]. Tabatabaei et al. declared that consolidations, air bronchogram, pleural effusion, and crazy paving are more common with admitted and deceased patients, which coincides with this study except for pleural effusion [6].

The study results also agreed with Parry et al. study which confirmed that crazy paving and consolidation were more commonly seen in clinically unstable patients [10]. The current study also went with Francone et al. who stated that GGO is more common in early cases unlike consolidation and crazy paving which are more common at late cases [11]. Similarly, Feng et al. reported that population with progressive disease showed crazy paving sign which reflects interstitial thickening [12].

This current study emphasized that confluent pattern of patches was more common with severe cases. And that matched with what was mentioned by Tabatabaei et al. [6] and Parry et al. [10] who found that peripheral lung involvement was seen in both stable and unstable patients but combined the central and peripheral distribution seen at the clinically unstable patients.

Nodules, septal thickening, and reticular densities were also noted in this study However, the other findings mentioned by Zheng Ye et al. [9] as halo sign, reversed halo sign, pleural effusion, and bronchiectatic changes were not seen. In the current study, nodules are most common with mild cases with good prognosis. This disagrees with what was reported at Silva et al. [13] study, which stated that this finding was associated with disease severity at admission and during hospital stay, and its detection ought to prevent any delay in medical treatment [13]. This could be explained by the different geographical and social distributions between the 2 studies which could affect clinical outcome. Guillo et al. found that subpleural lines were significantly prevalent in early phase, while fibrosis was seen at late phase [14]. This coincides with this work where fibrotic bands were significantly more incident in the markedly ill patients.

Zheng Ye et al. [9] and Haji Ahmadi et al. [7] stated that most of the cases showed lower lobar predominance. This was in contrast to what was stated in the current work that absent cranio-caudal predominance was the most common pattern.

The current study found significant relation between higher CT severity index and the poor clinical outcome. Similar results were found by Francone et al. who showed that progressive patients had greater lung involvement and higher CT severity scores [11].

## Conclusion

The current study concluded that CT chest is a good radiological marker that can help in predicting short-term clinical outcome in COVID-19 patient. We found that older patient’s age, consolidations, septal thickening, crazy paving, fibrotic bands, bilateral lung affection, mixed central and peripheral distribution, and higher CT severity scores are predictors of poorer clinical prognosis.

## Data Availability

Data are available at the data archiving system of Benha and Abbassia hospitals, which according to rules could not be shared.

## Abbreviations

COVID-19: Coronavirus disease 2019
RT-PCR: Reverse transcriptase polymerase chain reaction
GGO: Ground-glass opacities
CT: Computed tomography
CORAD: COVID-19 reporting and data system
